# Racemic epinephrine compared to salbutamol in hospitalized young children with bronchiolitis; a randomized controlled clinical trial [ISRCTN46561076]

**DOI:** 10.1186/1471-2431-5-7

**Published:** 2005-05-05

**Authors:** Joanne M Langley, Michael B Smith, John C LeBlanc, Heather Joudrey, Cecil R Ojah, Paul Pianosi

**Affiliations:** 1 Clinical Trials Research Centre, Dalhousie University, Halifax, Canada; 2 Departments of Pediatrics, Dalhousie University, Halifax, Canada; 3 Departments of Community Health and Epidemiology, Dalhousie University, Halifax, Canada; 4 Department of Pediatrics, Queen's University Belfast and Craigavon Area Hospital, Craigavon, Northern Ireland; 5 Department of Psychiatry, Dalhousie University, Halifax, Canada; 6 Saint John Regional Hospital, Atlantic Health Sciences Corporation, Saint John, Canada; 7 Division of Allergy, Immunology and Pulmonology, Department of Pediatric and Adolescent Medicine, Mayo Clinic Rochester, MN, USA

## Abstract

**Background:**

Bronchiolitis is the most common cause of lower respiratory tract illness in infancy, and hospital admission rates appear to be increasing in Canada and the United States. Inhaled beta agonists offer only modest short-term improvement. Trials of racemic epinephrine have shown conflicting results. We sought to determine if administration of racemic epinephrine during hospital stay for bronchiolitis improved respiratory distress, was safe, and shortened length of stay.

**Methods:**

The study was a randomized, double-blind controlled trial of aerosolized racemic epinephrine compared to salbutamol every one to 4 hours in previously well children aged 6 weeks to ≤ 2 years of age hospitalized with bronchiolitis. The primary outcome was symptom improvement as measured by the Respiratory Distress Assessment Instrument (RDAI); secondary outcomes were length of stay in hospital, adverse events, and report of symptoms by structured parental telephone interview one week after discharge.

**Results:**

62 children with a mean age of 6.4 months were enrolled; 80% of children had Respiratory Syncytial Virus (RSV). Racemic epinephrine resulted in significant improvement in wheezing and the total RDAI score on day 2 and over the entire stay (p < 0.05). The mean LOS in the epinephrine arm was 2.6 days (95% CI 2, 3.2) v. 3.4 days in those in the salbutamol group (95% CI 2.6, 4.2) (p > 0.05). Adverse events were not significantly different in the two arms. At one week post-discharge, over half of parents reported that their child still had a respiratory symptom and 40% had less than normal feeding.

**Conclusion:**

Racemic epinephrine relieves respiratory distress in hospitalized infants with bronchiolitis and is safe but does not abbreviate hospital stay. Morbidity associated with bronchiolitis as identified by parents persists for at least one week after hospital discharge in most infants.

## Background

Bronchiolitis accounts for up to 60% of all lower respiratory tract illness in the first year of life [1] and up to 32% of hospitalizations for lower respiratory tract illness in this age group [2]. The incidence of hospitalisation for bronchiolitis in infancy has increased in both the United States [3] and Canada [4] in the last two decades, and a 100% increase in first time hospitalisation for lower respiratory tract illness in children under two years has been noted in northern Europe [5].

Bronchiolitis is characterized by tachypnea and wheezing due to obstruction of small airways, and therefore treatment has often included use of beta and alpha agonists delivered by aerosol in addition to supportive care. A systematic review of randomised clinical trials of the efficacy of beta-agonist aerosols suggests they offer only modest short term improvement [6]. Alpha agonist stimulation of the sympathetic nervous system would be expected to reduce capillary leakage by constricting precapillary arterioles, reducing hydrostatic pressure and consequently bronchial mucosal edema [7]. Since Wohl and Chernick first suggested this intervention in 1978 [8], multiple studies and systematic reviews [9-11] have been published. While there is evidence that acute symptoms of bronchiolitis measured in the short term may improve with epinephrine, these reviews have called for more studies assessing longer-term outcomes such as duration of stay, and that are clinically relevant to parents, clinicians and the health care system.

We report a randomised controlled trial of aerosolised epinephrine compared to salbutamol throughout hospital stay in infants with bronchiolitis to assess daily clinical improvement (respiratory distress, feeding), length of hospital stay and adverse events, and outcomes by parental report one week after discharge to the community.

## Methods

This study was a randomized, double blind controlled trial of racemic epinephrine (Vaponefrin solution 2.25%, Aventis Pharma Inc, Laval, PQ) compared to control (salbutamol, Ventolin, GlaxoSmithKline Inc., Mississauga, ON) in children requiring hospitalization for management of bronchiolitis.

### Patient population

Eligible children were aged greater than 6 weeks to ≤ 2 years with a clinical diagnosis of bronchiolitis by the admitting physician. Wheezing had to be present on physical examination and was defined as a high-pitched, musical, continuous respiratory sound. Only patients admitted for management of bronchiolitis were eligible. The parent or guardian had to be able cooperate with study requirements (ability to speak, read and write English, have a telephone at home and not expected to move within the next month). The participating institutions were the IWK Health Centre in Halifax, Nova Scotia and the Saint John Regional Hospital (SJRH) in Saint John, New Brunswick. The IWK is a university-affiliated primary and tertiary-care pediatric hospital with an urban population of 300,000 and is a referral center for the Maritime provinces (population 2 million) of Canada. SJRH serves a rural-urban population of 200,000.

Children were not eligible for enrollment if they had had a previous diagnosis of asthma, were critically ill, or had chronic pulmonary or cardiac disease. Other exclusion criteria included: allergy to sodium metabisulfite, presence of tachycardia exceeding 200 beats per minute, or use of glucocorticoids, sympathomimetic amines or monoamine oxidase inhibitor therapy.

Informed consent was obtained from the parent or guardian prior to enrolment. The protocol was approved by the Ethics Review Board at both participating institutions.

### Study procedures

Study enrolment occurred in sequential winter respiratory seasons (November to April) from 1999 to 2002. Families were approached regarding study participation in the emergency department or within 24 hours of admission. Research nurses were available to enroll patients between 8 am and 8 pm.

Treatment allocation was determined by randomization, performed in blocks of four by the pharmacy department using a computer-generated random numbers table. Study drug was packaged in identical multidose vials labeled "study drug" with a code number. Both salbutamol and racemic epinephrine are clear, colorless liquids that are indistinguishable [12]. Participants were allocated to racemic epinephrine, 0.5 mls of 2.25% (Vaponefrin solution, Aventis Pharma, Montreal Quebec) or salbutamol respirator solution (Pharmel Inc., Montreal, Canada) by aerosol. Study drug was administered every one to four hours or more frequently at the request of the attending physician. Study drug was delivered by a wall flowmeter-nebulizer with face mask (Hospitak Inc., Farmingdale, NC) with oxygen at 5 to 7 L/min. A standard order sheet was used to ensure consistency of trial methodology. Salbutamol was given in 3 ml normal saline at a dosage of 1.5 mg for children weighing more than 10 kg, 1.25 mg for children >6 kg and < 10 kg, and 0.75 mg for those weighing less than 6 kgs. The heart rate was measured continuously during each aerosol and for one hour after. The heart rate, vomiting, presence of tremors or pallor were recorded in the health record by the bedside nurse at the end of every aerosol and one hour post aerosol.

### Data collection

Baseline demographic data collected at study entry included inclusion and exclusion criteria, age, gender, concomitant medications and other illnesses. The caregiver/parent was asked to describe the child's feeding pattern (normal, less than normal, unable to feed). At the time of study enrolment and then daily (every morning) thereafter the study nurse measured oxygen saturation and wheezing and retractions using the Respiratory Distress Assessment Instrument (Table 1) [13], which was the primary outcome measure of the study. Oxygen saturation was measured using a pulse oximeter (Nellcor Pulse Oximeter, Nellcor Puritan Bennet Inc., Pleasanton, CA) with the infant in a quiet state after breathing room air for at least 10 minutes. If the oxygen saturation went below 85%, the measurement was halted. At the daily assessment, the study nurse interviewed caregivers and reviewed the health record to determine if adverse events were present (vomiting, tremors, pallor), the feeding pattern and recorded the maximum daily heart rate for that 24-hour period.

**Table 1:** Respiratory Distress Assessment Instrument (From: Lowell DI, Lister G, Von Kloss H, McCarthy P. Wheezing in infants: the response to epinephrine. *Pediatrics *1987; 87:939-45.)

	0	1	2	3	4	Maximum Points
Wheezing						
Expiration	None	End	1/2	3/4	All	4
Inspiration	None	Part	All	...	...	2
Location	None	Segmental	Diffuse	...	...	2
						
Retractions						
Supraclavicular	None	Mild	Moderate	Marked	...	3
Intercostal	None	Mild	Moderate	Marked	...	3
Subcostal	None	Mild	Moderate	Marked		3
Total	...	...	...			17

* Within each variable (wheezing, retractions) the subscores are summed to give a total score. The maximum total points for wheezing is 8 and for retractions is 9.

During the first two study enrolments, it was noted that a bright red nasal discharge was observed in some study participants, and interpreted by bedside nurses as bloody nasal discharge. This discoloration of nasal mucous was found to be a known effect of administration of aerosolized epinephrine, which is caused by the oxidation of the sulphite stabilizer. This effect was not known to the investigators at the time of study design and is not in the drug monograph, but has been reported in a recent trial of epinephrine in the emergency department setting [14]. Because this could lead to unblinding of treatment allocation, an amendment to the study protocol was made for all subsequent patients whereby the bedside nurse wiped the nose of study participants after each study drug administration and immediately before the study nurse performed the daily respiratory assessment.

A nasopharyngeal aspirate for Respiratory Syncytial virus (RSV) antigen was routinely done in participating hospitals to determine appropriate placement for infection control purposes. At the discretion of the attending physician, some children had respiratory tract samples submitted for respiratory virus culture (RSV, influenza, parainfluenza, adenovirus).

A secondary outcome measure was duration of hospital stay, measured using a method previously validated by the Pediatric Investigators Collaborative Network on Infections in Canada studies of hospitalized children with RSV infection [15]. Each day the study nurse assessed which of four reasons accounted for ongoing hospitalization 1) patient receiving drug treatment for bronchiolitis 2) patient receiving oxygen supplementation or parenteral fluids because of bronchiolitis 3) patient hospitalized because of underlying (pre-existing) illness only or 4) awaiting transport home or uncertain home environment. Only those days on which the reason for hospitalization were one or more of receiving medication for bronchiolitis (1) or oxygen supplementation or parenteral fluids because of bronchiolitis (2), were recorded as valid hospital days. Discharge timing, counted as the time the decision was made to discharge home, was at the discretion of the attending physician. Study personnel had no involvement in discharge planning and did not impose any discharge criteria.

All parents/guardians were telephoned seven days after hospital discharge by a research assistant to collect data about the child's convalescence: respiratory symptoms (retractions, wheezing), feeding pattern (normal, less than normal, unable to feed), adverse events from medication (shakiness, tremors, pallor," other problems") and whether they had required a visit to a physician or to the emergency department or hospital. The interviewer read closed-ended questions from a standard script.

Adverse events were collected during the hospital stay and during the post-discharge telephone call. The event was described and categorized according to severity (mild, moderate, severe), outcome (recovered fully, recovered with sequelae, ongoing, death) and relationship to study drug (related, probably or possibly related, unrelated, unable to classify). Mild adverse events were defined as "awareness of signs and symptoms, easily tolerated and require no interventions", moderate as "discomfort sufficient enough to interfere with normal activities and/or result in some sort of intervention" and severe as " inability to perform normal activities, distressing and/or incapacitating and definitely require intervention and/or medical attention."

The sample size was calculated to detect a difference in the RDAI score between day one and day three. The estimated sample size for a two-sample comparison of proportions of each group that achieved the four-unit difference was with a probability of type one error of 0.05 and type 2 error of 0.8 was 33 infants per group. The standard deviations were based on previously reported changes in RDAI in bronchiolitis [13].

### Analysis

All randomized children were considered in the analysis. All analyses were performed using SAS 8.02 software (SAS Institute Inc., Cary, USA). Proportions and exact binomial intervals were calculated for discrete variables and comparisons between treatment groups were made using the Fisher's exact test. Summary statistics (mean, median, standard deviation, minimum and maximum) were calculated for continuous variables and comparisons were made between treatment groups using the Wilcoxon rank-sum test. Comparisons of trend across time were made using repeated measures analysis of variances. The RDAI was treated as a continuous measure. P-values less than or equal to 0.05 were considered statistically significant.

## Results

Sixty-two children were enrolled with 31 in each treatment arm. Ten were enrolled from the Saint John site and 52 from the Halifax site. All participants completed in-hospital follow-up but the parent of one child in the epinephrine group could not be contacted for the one-week post-discharge telephone call. The mean age of participants was 6.4 months. RSV was identified in nasopharyngeal samples of 81.5% of children randomized to epinephrine and 78.6% of those randomized on salbutamol. The two groups were similar at baseline; characteristics at enrolment are seen in Table 2. On admission, only 15% of infants had a normal feeding pattern; 83% had decreased feeding by parental/caregiver report and 7% were unable to feed. 245 children were screened who did not enroll: 46 did not meet inclusion criteria and in 34 parents refused consent. The rest were ineligible because of exclusion criteria. The most commons reason for exclusion was previous diagnosis of asthma (n = 58) and previous administration of systemic steroids (n = 44).

**Table 2:** Characteristics of children less than two years of age admitted to hospital with bronchiolitis by treatment group, at enrolment.

Outcome	Racemic Epinephrine	Salbutamol
Male	45.20 %	64.50 %
Age (median)	5.49 months	3.32 months
RDAI total	7.35 (95% CI 6.52, 8.19)	8.29 (95% CI 7.24, 9.34)
Wheezing	4.23 (95% CI 3.60, 4.85)	5.06 (95% CI 4.28, 5.85)
Retractions	3.13 (95% CI 2.64, 3.62)	3.23% (95% CI 2.71, 3.74)
		
Oxygen saturation (mean)	94.6 %	93.5 %
Abnormal feeding pattern (less than normal or not feeding)	90% ((95% CI 7.35, 97.9)	80% (95% CI 61.4, 92.3)
RSV positive	81.5% (61.9, 93.7)	78.6 (95% CI 59, 91.7)

RDAI = Respiratory Distress Assessment Instrument, CI = confidence interval

Racemic epinephrine resulted in a significant improvement in wheezing compared to salbutamol on day 2 (p = 0.01) and over the entire hospital stay (p = 0.01) (Table 3), but not on other days. The total RDAI (wheezing and retractions) in children receiving racemic epinephrine was also significantly better on the second hospital day and over the entire stay (p = 0.02). On the third hospital day a significant difference in oxygen saturation was observed in children receiving racemic epinephrine compared to those receiving salbutamol (96.20% v. 93.80%, 98.80 v. 92.00 p = 0.03) but this difference was not significant when the two groups were compared over the entire hospital stay or on other days.

**Table 3:** Respiratory Distress Assessment Instrument scores by hospital day in children with bronchiolitis randomized to aerosolized racemic epinephrine or salbutamol.

Hospital Day (Number of children in hospital for that duration)	Total Wheezing score		Retractions		Total RDAI score	
	Racemic epinephrine	Salbutamol	Racemic epinephrine	Salbutamol	Racemic epinephrine	Salbutamol
0 (n = 62)	4.23	5.06	3.13	3.23	7.35	8.29
1 (n = 58)	2.04	3.70	1.64	1.64	3.68	6
2 (n = 40)	1.59	2.65	1.06	1.06	2.65	4.13
3 (n = 22)	2.09	2.73	1.09	1.09	3.18	4.27
4 (n = 9)	2.75	4.00	1.75	1.75	4.5	6.4
5 (n = 9)	2.00	2.60	0.75	0.75	2.75	4.2

The mean length of stay for children randomized to the racemic epinephrine group was 2.60 days (95% CI 2.00, 3.20) and 3.40 days for those randomized to salbutamol (95% CI 2.60, 4.20) (p > 0.05). No significant differences in length of stay or RDAI scores were seen when children confirmed as having RSV infection were compared to those without RSV.

There was no difference in wheezing score, total retraction score, total respiratory score oxygen saturation or duration of stay by study site.

Any adverse event (mild, moderate, severe) was reported in 45.20% (14/31) of children who received epinephrine compared to 51.60% (16/31) of those who received salbutamol (p > 0.05). There was one severe adverse event (fever greater than 39°C rectal), judged unrelated to study medication, which occurred in a child on salbutamol. Tremors and pallor were more common in children receiving racemic epinephrine than in those on salbutamol, but these differences were not statistically significant (19.40% (6/31) v. 9.70% (3/31) and 19.40% (6/31) v. 6.50% (2/31) respectively). Vomiting occurred in 19.4% (6/31) of those receiving epinephrine and 25.8% (8/31) of those on salbutamol; this difference was not statistically significant.

At the follow-up telephone call, one week after discharge over 60% of infants had at least one ongoing respiratory symptom (Table 4) and most were still not considered to be feeding normally by their parents. No statistically significant differences in outcomes at the follow-up phone call were identified.

**Table 4:** Prevalence of respiratory symptoms and drug-related adverse events one week after discharge in children randomized to aerosolized racemic epinephrine or salbutamol during their hospital stay for bronchiolitis.

Clinical event as reported by parent or guardian	Percentage of children with symptom at one week post-discharge phone call (absolute numbers)*		P value for comparison of proportions
	Racemic epinephrine	Salbutamol	
Breathing difficulty	20.00 (6/30)	19.4 (6/31)	1.00
Wheezing	56.7 (17/30)	67.7 (21/31)	0.43
Chest retractions	13.3 (4/30)	25.8 (8/31)	0.33
Feeding pattern:			
Normal feeding	50 (15/30)	54.8 (17/31)	0.80
Less than normal	50 (15/30)	41.9 (13/31)	0.61
Unable to feed	0 (0/31)	3.2 (1/31)	1.00
Vomiting	20 (6/30)	35.5 (11/31)	0.25
Tremors	0 (0/31)	0 (0/31)	N/A
Pallor	13.3 (4/30)	6.5 (2/31)	0
Has had a visit to a physician	23.3 (7/30)	19.4 (6/31)	0.42
Has visited an emergency department or been hospitalized	3.3 (1/30)	3.2 (1/31)	1.00

N/A = not applicable; * Not mutually exclusive

## Discussion

In this study, treatment of bronchiolitis with aerosolised racemic epinephrine over the course of a child's hospital stay was associated with improvement in respiratory symptoms, but did not result in a statistically significant difference in hospital length of stay. Although previous reports have found that nebulized epinephrine results in short-term clinical improvement in bronchiolitis [12,16-21], its effect on duration of hospital stay or need for admission is less clear. Two of four randomised clinical trials in the emergency department setting using 1 to 3 doses of epinephrine have found a difference in admission rate [12,19] and two have not [22,23]. It is possible that small reductions in length of stay could be detected by larger trials than those conducted thus far. Our study is only the second [24] in which nebulized racemic epinephrine was provided throughout the hospital admission. Other trials have administered one to three doses spaced on one day only. One might expect that short-term improvement could lead to the clinician's judgement that admission was not necessary. Two [20,21] of four randomised trials [24,25] have suggested that length of stay in hospital is abbreviated in children receiving epinephrine. The subgroup of children that might benefit from this therapy is not clear and larger trials will be necessary to identify if and when administrations of nebulized epinephrine during hospitalisation results in patient benefit. Our study supports the thesis that while airway edema may be improved following administration of racemic epinephrine it is not sustained and does not alter the natural history of bronchiolitis in infants. The inflammatory process initiated by RSV or other respiratory viruses is unaffected and mucous secretion and edema recurs after the effect of epinephrine has dissipated.

We choose salbutamol as a control for epinephrine because it was the local standard of care at the time our trial was designed and it was considered unethical to withhold a potentially beneficial therapy, even though that benefit was likely minimal [6]. Other randomized trials of epinephrine in hospitalized children have used as the control normal saline [22,25], salbutamol [21] or both [24].

Interestingly we noted a risk to unblinding of treatment allocation early in our trial, by the oxidation of the sulphite preservatives, which turn mucous red or brown in recipients of racemic epinephrine. We implemented measures to avoid unblinding, but could find no mention of this event in previous studies of epinephrine in bronchiolitis. If future trials of racemic epinephrine are conducted, study design should carefully address this possibility and consider using another formulation of epinephrine. Patel et al questioned study personnel and ward staff after completion of their trial and found no difference in the proportion of correct guesses as to allocation by treatment group [24].

Hospital admission for bronchiolitis occurs when the infant has significant respiratory distress or is unable to feed because of the work of breathing. By this point in the evolution of bronchiolitis, lower respiratory tract inflammation is well established and may be difficult to alter. Perhaps for this reason, trials of steroid therapy in infants hospitalized for bronchiolitis have shown no benefit [11]. Length of hospital stay or avoidance of hospital admission is a salient outcome measure for intervention trials of epinephrine because institutional care represents the largest component of direct expenditures (over 60%) for bronchiolitis [26]. Morbidity associated with bronchiolitis persisted for at least one week after hospital discharge in our population. Reducing costs and morbidity due to bronchiolitis may require more than one intervention, each at specific times during the illness. For example Schuh et al demonstrated a reduction in hospitalization in infants with bronchiolitis treated with dexamethasone (1 mg/kg) in the emergency department [27]. Bisgaard et al demonstrated a reduction in post-RSV symptomatology, principally cough, in infants treated with montelukast within the first week of illness [28]. All of these treatments, if their efficacy were borne out in larger trials, would reduce health care costs associated with this ubiquitous infection. Such studies will need to be large to capture clinically significant outcomes.

Use of racemic epinephrine multiple times over several days was not associated with significant adverse events compared to salbutamol. Epinephrine is a potent adrenergic agonist with potential cardiovascular side effects including tachycardia or bradycardia and hypertension. In the doses used for bronchiolitis such adverse events have not been reported. Given that short-term improvement may occur and its favourable safety profile, it seems reasonable to use aerosolized racemic epinephrine selectively for infants with acute distress to decrease the work of breathing or to avoid assisted ventilation.

## Conclusion

Racemic epinephrine relieves respiratory distress (wheezing, retractions) in infants hospitalized for management of bronchiolitis and is safe but does not abbreviate hospital stay. Morbidity associated with bronchiolitis as identified by parents persists for at least one week after hospital discharge in most infants.

## Abbreviations

Respiratory Distress Assessment Instrument (RDAI)

Respiratory Syncytial Virus (RSV)

## Competing interests

The author(s) declare that they have no competing interests.

## Authors' contributions

JML and MBS conceived the study and prepared the protocol. JCL planned the statistical analysis. JCL and PP provided intellectual input to study design. JML drafted the manuscript. JML, MBS and CBO supervised acquisition of study data. HJ conducted the analysis. All authors contributed to interpretation of study results and critically reviewed the manuscript for important intellectual content.

## Pre-publication history

The pre-publication history for this paper can be accessed here:


